# Postoperative malignant hyperthermia confirmed by calcium-induced calcium release rate after breast cancer surgery, in which prompt recognition and immediate dantrolene administration were life-saving: a case report

**DOI:** 10.1186/s13256-021-02681-0

**Published:** 2021-04-17

**Authors:** Natsumi Miyazaki, Takayuki Kobayashi, Takako Komiya, Toshio Okada, Yusuke Ishida, Hidekimi Fukui, Yukihiko Ogihara, Hiroyuki Uchino

**Affiliations:** 1 Department of Anesthesia, Tokyo Medical University, 6-7-1 Nishi-shinjuku, Shinjuku-ku, Tokyo 160-0023 Japan; 2 Department of Plastic Surgery, Tokyo Medical University, 6-7-1 Nishi-shinjuku, Shinjuku-ku, Tokyo Japan

**Keywords:** Malignant hyperthermia, Postoperative hyperthermia, Calcium-induced calcium release rate (CICR)

## Abstract

**Background:**

Malignant hyperthermia (MH) is a rare genetic disease characterized by the development of very serious symptoms, and hence prompt and appropriate treatment is required. However, postoperative MH is very rare, representing only 1.9% of cases as reported in the North American Malignant Hyperthermia Registry (NAMHR). We report a rare case of a patient who developed sudden postoperative hyperthermia after mastectomy, which was definitively diagnosed as MH by the calcium-induced calcium release rate (CICR) measurement test.

**Case presentation:**

A 61-year-old Japanese woman with a history of stroke was hospitalized for breast cancer surgery. General anesthesia was introduced by propofol, remifentanil, and rocuronium. After intubation, anesthesia was maintained using propofol and remifentanil, and mastectomy and muscle flap reconstruction surgery was performed and completed without any major problems. After confirming her spontaneous breathing, sugammadex was administered and she was extubated. Thereafter, systemic shivering and masseter spasm appeared, and a rapid increase in body temperature (maximum: 38.9 °C) and end-tidal carbon dioxide (ETCO_2_) (maximum: 59 mmHg) was noted. We suspected MH and started cooling the body surface of the axilla, cervix, and body trunk, and administered chilled potassium-free fluid and dantrolene. After her body temperature dropped and her shivering improved, dantrolene administration was ended, and finally she was taken to the intensive care unit (ICU). Body cooling was continued within the target range of 36–37 °C in the ICU. No consciousness disorder, hypotension, increased serum potassium level, metabolic acidosis, or cola-colored urine was observed during her ICU stay. Subsequently, her general condition improved and she was discharged on day 12. Muscle biopsy after discharge was performed and provided a definitive diagnosis of MH.

**Conclusions:**

The occurrence of MH can be life-threatening, but its frequency is very low, and genetic testing and muscle biopsy are required to confirm the diagnosis. On retrospective evaluation using the malignant hyperthermia scale, the present case was almost certainly that of a patient with MH. Prompt recognition and immediate treatment with dantrolene administration and body cooling effectively reversed a potentially fatal syndrome. This was hence a valuable case of a patient with postoperative MH that led to a confirmed diagnosis by CICR.

## Background

Malignant hyperthermia (MH) is a very rare disease, in which symptoms are induced by a pharmacogenomic reaction, caused by genetic mutations in the ryanodine receptor 1 (RYR1) gene, which encodes a calcium channel protein. The prevalence of MH is 1 in 100,000–150,000 patients who are administered anesthetics [1]. MH is induced mainly by volatile anesthetics or musle relaxants, and its symptoms include muscle rigidty, high fever, acidosis, tachycardia, and rhabdomyolysis. Without prompt and appropriate treatment, symptoms become life-threatening. In Japan, more than 400 cases of the fulminant type of MH have been reported from 1960 to the present. The sex difference is 3:1 (men/women). However, postoperative MH is very rare, representing only 1.9% of all suspected MH cases as reported in the North American Malignant Hyperthermia Registry (NAMHR) [2]. Here we report a case of a patient who developed sudden postoperative hyperthermia after mastectomy, which was definitively diagnosed by the postoperative calcium-induced calcium release rate (CICR) measurement test.

## Case presentation

The patient was a 61-year-old Japanese woman, who was admitted to our hospital for breast cancer surgery. She had a medical history of stroke and regularly took aspirin. Her height was 163 cm and her body weight was 47 kg. She had no history of allergies, no relevant social history, no muscle disorders, no family history of MH, and no abnormal findings on preoperative examination.

**Fig. 1:**
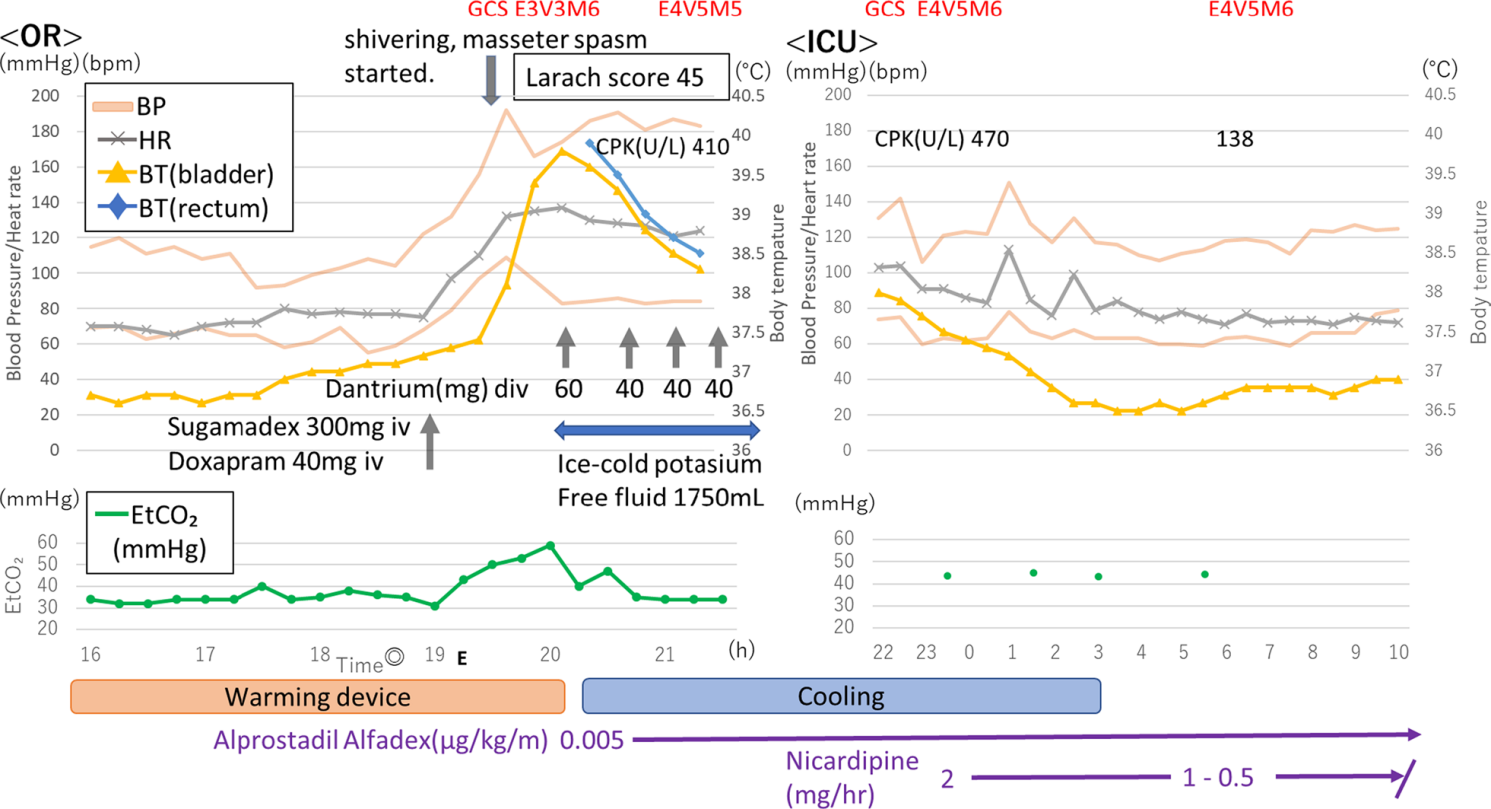
Time course of anesthetic management in the operating room and during stay in the intensive care unit. *Larach score: clinical grading scale for MH (◎ = completion of the operation; E = extubation)

General anesthesia was introduced by intravenous injection of propofol (target-controlled infusion: TCI 4.5 µg/mL), remifentanil (0.35 µg/kg/min), and rocuronium (40 mg). After intubation, anesthesia was maintained by propofol (TCI 2.5 µg/mL) and remifentanil with bispectral index monitoring, and mastectomy and muscle flap reconstruction surgery was performed. The surgery itself was completed without any major problems. Operation time was 560 minutes, and anesthesia time was 811 minutes. Figure 1 shows the time course of anesthetic management in the operating room and during her stay in the intensive care unit (ICU).

The time course for core body temperature, blood pressure, heart rate, EtCO_2_, Glasgow Coma Scale (GCS), creatinine phosphokinase (CPK), and Larach score, which is used for the clinical grading of MH [6], is shown in Fig. 1. During the operation until the time of ICU admission, we also administered alprostadil alfadex to control the patient’s blood pressure and to maintain blood perfusion for the muscle flap reconstruction.

After the operation and during recovery from anesthesia, the patient showed weak spontaneous respiration and recovery of muscle power, and responded to our instructions but was still slightly drowsy. We observed her for about 15 minutes; however, her recovery was not sufficient. Therefore, we finally decided to administer sugammadex (300 mg) and doxapram (40 mg) to antagonize the effects of the muscle relaxant and anesthetic agents. The patient subsequently showed good recovery of consciousness and respiration. We extubated the patient, and 15 minutes later, shivering and masseter spasm were observed, as well as increases in blood pressure and body temperature (maximum: 39.7 °C) and EtCO_2_ (maximum: 59 mmHg). To measure ETCO_2_, we put a capnometer on the patient’s mouth and sealed it using an oxygen mask. We also approximated partial pressure of carbon dioxide (PaCO_2_) from blood artery gas measurements. At the same time, her consciousness level became drowsy again (GCS: E3V3M5). Blood gas analysis (FiO_2_, 1.0) demonstrated the following: pH 7.279, paCO_2_ 59 mmHg, partial pressure of oxygen (pO_2_) 37.8 mmHg, bicarbonate anion (HCO_3_^−^) 26.8 mmol/L, base excess (BE) 0.8, hemoglobin (Hb) 11.8 g/dL, hematocrit (Hct) 36.4%, sodium ion (Na^+^) 146 mmol/L, potassium (K) 3.9 mmol/L, chloride anion (Cl^−^) 113 mmol/L, calcium ion (Ca^2+^) 1.24 mmol/L, glucose (Glu) 124 mg/dL, and lactate (Lac) 1.8 mmol/L. We suspected postoperative MH and started continuous body cooling, and the administration of cold potassium-free fluid (total: 1750 mL) and dantrolene (3.6 mg/kg; total: 180 mg) to prevent the loss of urine and reduce the body temperature to within the target range of 37–38 °C 40 minutes after the extubation, and by 90 minutes after the extubation, we succeeded in reducing the body temperature to approximately 38.1 °C. We then stopped the administration of dantrolene. The patient’s hypertension did not resolve upon the administration of antihypertensive drugs, but her consciousness level became clear (Glasgow Coma Scale [GCS]: E4V5M6).

The patient was transferred to the ICU for continuous cooling and observation. Continuous infusion of a calcium channel blocker was started. The patient’s maximum creatine phosphokinase (CPK) level was 470 U/L. No consciousness disorder, hypotension, increased serum potassium, metabolic acidosis, cola urine, or acute kidney or liver injury was observed during her ICU stay. The patient was discharged to the general ward on postoperative day 1 (POD1) without any complications.

Muscle biopsy (Calcium-induced calcium release [CICR] measurement test) was performed after the patient’s discharge, providing a definitive diagnosis of MH. As there are only two facilities in Japan at which CICR measurements can be performed, we were unable to perform the measurements immediately at the time. However, genetic analyses for mutations in the ryanodine receptor (RYR1) and voltage-dependent Ca channel (dihydropyridine receptor [DHPR]) genes were not performed.

## Discussion

MH is an abnormal state of hypermetabolism of the skeletal muscle. It is caused by mutations in RYR1 and DHPR, which are located in the sarcoplasmic reticulum (SR) of the skeletal muscle [2]. In MH, abnormal and uncontrollable intracellular calcium release in the skeletal muscle induces a hypermetabolic state in the muscle, leading to hyperthermia, acidosis, and rigidity. In most cases, volatile inhaled anesthetics induce CICR from the SR, and in particular, our patient with postoperative MH triggered by propofol anesthesia is a very unusual case. Visoiu *et al*. reported 477 MH cases that met the inclusion criteria, among which 58.5% were possible MH and 41.5% were fulminant MH. Inhaled anesthetics and succinylcholine had been administered in 53.9% of cases, inhaled anesthetics in 41.7%, and succinylcholine without inhaled anesthetics in 2.9%. No causative anesthetic drugs were reported in seven MH cases [3]. Signs of MH often appear at later time points, in the second or third hour of anesthesia [3]. The frequency of MH occurrence is extremely rare, being observed in 1 in 50,000–150,000 general anesthesia cases, and analysis of the RYR1 gene has indicated that approximately 1 in 2000 people have a predisposition to MH [3]. The occurrence of MH may be life-threatening, and genetic testing and muscle biopsy are required for a definite diagnosis. However, postoperative MH, which is generally observed within less than an hour after discontinuation of the anesthetic agent, is very rare, representing only 1.9%, of cases, as reported by Litman *et al*. [4]. Postoperative delayed MH after off-pump coronary bypass surgery has been reported [5].

Our patient showed an initial rise in body temperature about 40 minutes after the discontinuation of anesthesia, which was consistent with NAMHR in terms of onset [3]. Although shivering and masseter spasm without hyperthermia were observed just after extubation, it was very difficult to diagnose MH at this point because of the nonspecific nature of the clinical signs and symptoms of MH.

**Fig. 2:**
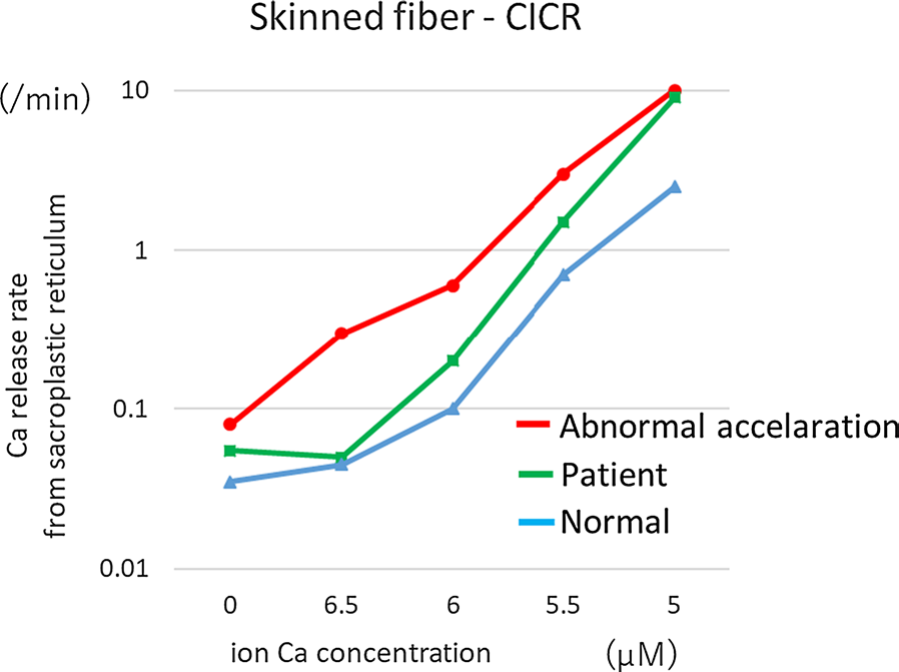
A graph of calcium-induced calcium release (CICR) performed after discharge. Horizontal axis shows calcium ion concentration (µM), and vertical axis shows the rate of calcium release from the sarcoplasmic reticulum. The pattern is similar to that of abnormal acceleration (green)

It is very important for anesthesiologists to diagnose unusual situations as early as possible to avoid complications and mortality associated with MH. Larach *et al*. reported that cardiac dysfunction and change in consciousness level were the most common MH-associated complications, and they also reported that the clinical grading scale (CGS) was useful as clinical diagnostic criteria for MH [6]. Retrospective evaluation of the clinical features of our present patient by CGS resulted in a score of 45, which suggests that this patient almost certainly had MH (Fig. 1). The most reliable test to confirm the diagnosis of MH is muscle biopsy and CICR using the skinned fiber method, in which the muscle fibers are unsheathed by removing the cell membrane [7]. Fortunately, we were able to obtain informed consent for muscle biopsy from the patient. Figure 2 shows the results of the CICR test performed after discharge, which reveals a pattern similar to abnormal acceleration. Therefore, we were able to confirm that this patient had MH. This is a valuable case of a patient with postoperative MH, in which the diagnosis was confirmed by CICR. We used propofol, a non-depolarizing muscle relaxant (rocuronium bromide), and opioids (fentanyl and remifentanil) for anesthetic management. These agents can be safely administered to malignant hyperthermia-susceptible (MHS) patients [8]. Although the cause of MH in our patient is unclear, we considered the possibility of sugammadex or other drugs. We administered a total of 6 mg/kg sugammadex before the patient showed symptoms of MH. Although we initially antagonized the effects of the muscle relaxants using 4 mg/kg sugammadex, the patient showed insufficient recovery, so we administered an additional 2 mg/kg sugammadex. The patient then achieved sufficient recovery from the muscle relaxant. However, we believe that the use of train-of-four (TOF) monitoring to evaluate the recovery from the muscle relaxant would have been useful. It is possible that our patient was affected by other volatile anesthetics (sevoflurane or desflurane) used in a previous operation in the same room, because the anesthetic circuit was not sufficiently washed out. We also administered doxapram to reverse the residual effects of the anesthetic agents [9]. However, it has been reported that doxapram can be used for MH patients, so agents that induce postoperative MH are unknown. We quickly warmed the patient using a warming system (3M™ Bair Hugger™), because she was hypothermic (35.8 °C) at the end of the operation. Monish *et al.* suggested that exercise in excessive heat can trigger MH [7]. A core temperature of less than 36 °C should be maintained during the postoperative period in MHS patients [10].

Dantrolene is a specific drug recommended for the treatment of MH. Some reports recommend a dose of 2.5 mg/kg every 5 minutes until clinical improvement is seen [11, 12]. These reports also suggest that continuous administration of dantrolene (1 mg/kg every 4–6 hours) should be performed for 24–48 hours after the operation.

Riazi *et al*. reported that a longer than 50-minute delay in the administration of dantrolene increased the rate of complications to 100% [13]. We administered a total of 3.6 mg/kg dantrolene beginning 30 minutes after the patient’s hyperthermia started, and succeeded in effectively counteracting the hyperthermia.

To control hypertension and to maintain comfortable tissue perfusion, we selected alprostadil alfadex for use during the operation rather than a calcium channel blocker. After the patient was transferred to the ICU, we combined this with the administration of a calcium channel blocker as well. The patient showed hypercarbia and hyperthermia, but not electrolyte abnormalities, arrhythmia, or myoglobinuria owing to rhabdomyolysis. Aggressive fluid replacement with crystalloids was able to produce sufficient amounts of urine to reduce the risk of myoglobin precipitation in the renal tubules [8], and dantrolene administration improved the abnormal hypermetabolism of the skeletal muscle, leading to the correction of hypercarbia and hyperthermia.

Regarding the avoidance of anesthetic triggers, a rapid diagnosis of MH and the prompt use of dantrolene is expected to lead to a reduction in complications and mortality owing to MH. Here we reported the case of a patient with postoperative MH, which is very rare. However, rapid recognition and treatment with dantrolene proved to be life-saving.

## Conclusions

The occurrence of MH may be life-threatening, but its frequency is very low, and genetic testing and muscle biopsy are required for a definitive diagnosis. In the retrospective evaluation using the malignant hyperthermia scale, it was concluded that this patient almost certainly had postoperative MH. Prompt recognition and immediate treatment with intravenous administration of dantrolene and body cooling effectively reversed the symptoms of this potentially fatal syndrome. This was a valuable case of a patient with postoperative MH, in which the diagnosis was confirmed by CICR.

## Data Availability

Not applicable
